# An Approach to Investigate Content-Related Quality of Nutraceuticals Used by Slovenian Consumers: A Case Study with Folate and Vitamin D Supplements

**DOI:** 10.3390/foods10040845

**Published:** 2021-04-13

**Authors:** Katja Žmitek, Sanja Krušič, Igor Pravst

**Affiliations:** 1 Nutrition Institute, Tržaška Cesta 40, SI-1000 Ljubljana, Slovenia; katja.zmitek@vist.si (K.Ž.); sanja.krusic@nutris.org (S.K.); 2 VIST–Higher School of Applied Sciences, Gerbičeva Cesta 51A, SI-1000 Ljubljana, Slovenia; 3 Biotechnical Faculty, University of Ljubljana, Jamnikarjeva 101, SI-1000 Ljubljana, Slovenia

**Keywords:** food supplements, dietary supplements, public health, safety, quality, vitamin D, cholecalciferol, folate, folic acid

## Abstract

A predisposition for the efficiency of nutraceuticals is that the product contains a sufficient quantity of a vitamin. Several studies have highlighted different quality issues. Our objective was to investigate whether the contents of the vitamins in selected types of food supplements were in accordance with labeling. We focused on two types of food supplements where content-related quality issues could result in public health risks: food supplements for supplementation with (a) folic acid (as 5-methyltetrahydrofolate (5-MTHF)) in pregnancy and (b) with vitamin D in the general population. The study was done on supplements from the global supply that are typically used by Slovenian consumers. We sampled one production batch of 30 different food supplements—six and 24 samples with 5-MTHF and cholecalciferol, respectively. We found samples with vitamin contents outside the 80–150% tolerance interval in both sets. Particularly, 5-MTHF was found to be more problematic, probably due to its lower stability. This study shows the need for better quality control. Quality control is needed during both the manufacturing process and product shelf lifetimes. Content quality should be also subject to external controls by authorities. Voluntarily quality control schemes would also enable consumers to identify products of sufficient quality.

## 1. Introduction

Nutraceuticals present a common strategy for combating nutritional deficiencies and fighting aging-related pathologies. While there are considerable regional differences, about 50% of the adult population regularly uses food supplements in some countries [1,2]. Typically, the usage of supplements is higher in women than in men, while vitamins and minerals (and their combinations) are reported as the most commonly consumed supplements [1].

In the European Union (EU), food supplements are regulated as foods, and their quality is under the responsibility of food business operators (e.g., producers and distributors) [3,4,5]. The regulation defines food supplements as “foodstuffs, the purpose of which is to supplement the normal diet and which are concentrated sources of nutrients or other substances with a nutritional or physiological effect” [3]. Typically, these are marketed in the form of tablets, capsules, drops, sprays, or other small dosages. The goals of the harmonization of the regulation in 2002 were to support the functioning of the internal EU market (free movement of goods, unequal conditions of competition, etc.) and to protect consumers against misleading practices and potential health risks [3,5]. While businesses dealing with food supplements in the EU need to comply with Hazard Analysis Critical Control Points (HACCPs), there is no requirement for good manufacturing practices (GMPs). The fact that quality standards and registration costs for food supplements are not comparable with medicinal products should be reflected in lower manufacturing expenses. In theory, this should result in more affordable (cost-effective) products of sufficient quality for supplementing normal diets. Additionally, because food supplements are not labeled with detailed warnings and possible adverse effects (which are required for medicines), consumers can perceive them as safer for use. Paradoxically, concerns regarding the quality of food supplements in Europe have been raised, suggesting the need for a stricter regulatory environment [6].

When discussing quality issues for food supplements, different dimensions of quality need to be mentioned. Quality issues can be assigned to either unintentional (e.g., due to a lack of quality control, instability, and contamination) or intentional (e.g., adulteration, with the motivation of economic gain) acts [7]. Typical examples of quality issues are (a) mislabeling [6], (b) the presence of contaminants [8], (c) adulterations and usage of unauthorized substances [9,10,11], and (d) active ingredient content quality issues [12,13,14,15,16,17]. For example, Mannino et al. [17] recently investigated 24 food supplements that claimed to contain cranberry, but 17 samples did not comply with the uniformity test of dosage forms and only five actually contained cranberry. The majority of samples also claimed an incorrect amount of bioactive compounds. Furthermore, Gaspar et al. [16] reported an unacceptable quality of several food bilberry supplements. Some did not even contain bilberry, and some samples were falsified with anthocyanins from other sources. Content quality issues were also reported for supplements containing essential elements [12], coenzyme Q10 [13], carotenoids [14,15], and vitamins [18,19,20,21,22]. 

While, in some cases, quality issues could be interpreted as the misleading of consumers, these sometimes present not only safety risks for individual users but also wider public health threats [7]. It should be noted that food supplements are complex products, and that their quality is dependent not only on the quality of ingredients but also on the whole manufacturing process. Supplements usually contain a variety of very sensitive constituents that need to be stabilized with appropriate processing techniques. This can be a major technological challenge for many manufacturers [23]. Quality control is also crucial for both production and shelf life; however, the laboratory quantification of certain constituents can be challenging [24,25,26,27,28]. The bioavailability of ingredients used in food supplements and their interactions also need to be considered, but this area is commonly overlooked and very challenging to regulate [29].

While the presence of contaminants and unauthorized substances in food supplements should obviously be regarded as a safety issue, the situation is less clear for active ingredient content quality issues. For example, according to the European Commission (EC) guidance document [30], the acceptable content of most vitamins is in the range of 80–150% of the declared content. It should be noted that this uncertainty includes all factors for variations, including measurement uncertainty [30]. Not meeting this content-related quality criteria is of course misleading for consumers, and, in some situations, it can also present a health risk. This can occur when food supplements are marketed to those with nutritional deficiencies or other medically relevant needs for dietary supplementation. We identified two types of food supplements where this is particularly relevant, namely those intended for supplementation with folic acid in pregnant women and those intended for supplementation with vitamin D in the populations with a high prevalence of deficiency.

It is well-established that the supplementation of a standard diet with 400 μg of folic acid decreases the risk of neural tube defects by 50% [31,32], and supplementation is therefore recommended to women of child-bearing age from at least one month before to at least three months after conception. Women supplement their diets with the intent to reduce health risks; thus, it is particularly important that they administer the correct amount of folic acid. Failure to do so (i.e., if they were to take folic acid pills with insufficient amounts of folic acid) would not only mislead the consumer but also be a safety risk. It should also be noted that, in the liver, folic acid is transformed into active 5-methyltetrahydrofolate (5-MTHF), which is the donor of a methyl group in epigenetic processes of embryogenesis and many other processes [33]. In specific circumstances, supplementation with 5-MTHF can be more effective [33], and this argument is sometimes used for the aggressive marketing of 5-MTHF supplements to pregnant women, which can be used as an alternative to subscription medicines containing folic acid.

Another relevant example involves food supplements intended for supplementing vitamin D. In many countries, an extremely high prevalence of vitamin D deficiency has been reported, particularly during winter periods [34,35,36], due to seasonal variations in the efficiency of the ultraviolet B (UVB)-induced biosynthesis of vitamin D in human skin [37]. In Slovenia, for example, about 80% of the adult population was found to have insufficient vitamin D levels between October and April [35]. Though there are differences between countries, the recommended daily intake of vitamin D for adults (in the absence of endogenous synthesis) is usually between 15 and 20 µg [38,39]. Considering the well-established role of vitamin D in human health [40], it is particularly important that products intended for the administration of vitamin D in populations with a nutrient deficiency contain a sufficient amount of this vitamin.

However, the data on the content-related quality of food supplements in Europe are limited. Our goal was therefore to investigate whether the content of the active ingredient in selected types of food supplements on the market is in accordance with the content provided by manufacturers on the labeling. We focused on two types of food supplements where content-related quality issues could result in public health risks: (a) food supplements for supplementation with folic acid in pregnancy and (b) those for supplementation with vitamin D in the general population with a high prevalence of deficiency. While this case study investigated samples of food supplements particularly relevant for Slovenian consumers, the vast majority of samples are international brands that are available across the EU marketplace. A targeted sampling protocol was used to assure the selection of samples from a wide range of manufacturers.

## 2. Materials and Methods

### 2.1. Sample Selection

#### 2.1.1. Food Supplements for Supplementation with Folate in Pregnancy

We focused on food supplements marketed for folate supplementation in pregnancy, with folate in the form of active 5-MTHF. To identify products easily available to Slovenian consumers, screening was done using the government-authorized online pharmacy Lekarna Nove Poljane (Ljubljana, Slovenia; URL: www.lekarnar.com; accessed on 20 September 2020). A search using terms “folic acid” and “pregnancy” (note: search was done in Slovenian language) resulted in 18 available food supplements. Of these, 10 supplements met the inclusion criteria because they contained folate in the 5-MTHF form. Four products were excluded because they presented either a different packaging size of same product (*N* = 1) or a very similar product from the same producer (*N* = 3). The final sample was composed of six food supplements. All selected food supplements (*N* = 6) were purchased in October 2020, verified to be within the usage shelf life as defined by the manufacturer, and sent to the laboratory for analyses. Samples contained folate in the form of either (a) calcium-l-methylfolate or (b) glucosamine salt of (6*S*)-5-methyltetrahydrofolic acid; both are approved for use in food supplements in the EU [3]. All products were international brands marketed across the EU, none of which were produced in Slovenia.

#### 2.1.2. Food Supplements for Supplementation with Vitamin D in the General Population

We focused on food supplements marketed for vitamin D supplementation in the general population. We first tested a previously described approach for the selection of supplements with 5-MTHF (Section 2.1.1), but a search with the term “vitamin D” resulted in *N* = 138 different food supplements in the online pharmacy. This was above our capacity and therefore not feasible. Therefore, we decided to first conduct a survey on Slovenian consumers to identify most commonly used vitamin D supplements.

An online survey was developed using the SurveyMonkey platform (SurveyMonkey Europe UC, Dublin, Ireland), and a press release was launched by the Nutrition Institute with an invitation to those supplementing vitamin D so that they could provide details about used products. Slovenian mass media then communicated this invitation in printed, aired, and online formats. An invitation to the survey was also published on the Facebook page of the Nutrition Institute. The survey was anonymous (without any personal data); however, multiple usage from the same browser was restricted. The inclusion criterion was regular supplementation with vitamin D. The survey had four questions. In the first question, subjects were asked if they were taking vitamin D via medicines (all registered vitamin D drugs were specified) or food supplements. If the subject selected food supplements, the next question was to select the formulation type of the supplement (capsules, tablets, spray, drops, chewable or effervescent tablets, or other), followed by the provision of details of the specific food supplement used for supplementation with vitamin D. Subjects were asked to provide as many details as possible to enable the identification of the product. Altogether, we received *N* = 688 valid survey responses, reporting the use of 95 different food supplements.

A decision was taken to include food supplements that (a) had at least three reported users in our survey, (b) were labeled to contain 10–100 µg of vitamin D, (c) were from different manufacturers, and (d) were not multicomponent supplements. Altogether, *N* = 34 products were reported by at least three reported users. After the implementation of other above-mentioned inclusion/exclusion criteria, the final sample was *N* = 24. We should note that if one manufacturer offered more supplements with different dosages of vitamin D, we sampled the one with 20 µg of vitamin D, as this was most prevalent vitamin D dosage. If this was not possible (e.g., such product did not exist or was out of stock), we sampled the product with the highest reported usage in our survey. All the selected food supplements (*N* = 24) were purchased in November/December 2020, verified to be within the usage shelf lives as defined by the manufacturers, and delivered to the laboratory for analyses. The labeling of all the purchased samples was also checked for the sources of vitamin D. Though both cholecalciferol and ergocalciferol are approved for use in food supplements in the EU [3], all the sampled products contained vitamin D in the form of cholecalciferol. The majority of the products were international brands that are marketed across the EU.

### 2.2. Quantification of the Active Ingredient 

To enable the use of study result by food control authorities, quantifications of active ingredients were conducted by an out-sourced accredited laboratory. 5-MTHF was measured using the accredited liquid chromatography–mass spectrometry (LC–MS/MS) method SOP M3816 [41], and cholecalciferol was measured using the accredited LC–MS/MS method MP 1570 rev 2/2017 [42]. The limits of quantification were 0.25 μg/100 g for 5-MTHF and 20 µg/100 g for cholecalciferol. Analytically determined content was provided per dosage (e.g., per pill or per spray). For liquid formulations, density was also measured.

### 2.3. Data Analyses and Conformity with Regulatory Requirements 

The results of laboratory analyses were compared with the labeled contents of the vitamin in the food supplements. For each sample, we calculated the analytically determined percentage (%) of the labeled vitamin content as follows:

$$\left(\%\right)\;\mathrm{labeled}\;\mathrm{vitamin}\;\mathrm{content}=\frac{\mathrm{analytically}\;\mathrm{determtextned}\;\mathrm{content}\;\mathrm{of}\;\mathrm{vitamin}}{\mathrm{content}\;\mathrm{provided}\;\mathrm{by}\;\mathrm{manufacturer}\;\mathrm{on}\;\mathrm{labeling}}$$

Data were processed and analyzed using Microsoft Excel 2019 (Microsoft, Redmond, Washington, DC, USA). Descriptive analysis was used to report the proportion of conformity with legal requirements. Samples with an analytically determined vitamin content within the range of 80–150% of the labeled value were assigned as compliant with regulatory requirements according to the EC guidance document [30].

## 3. Results and Discussion

### 3.1. Food Supplements for Supplementation with Folate in Pregnancy

We sampled six (*N* = 6) 5-MTHF-containing food supplements that are marketed for supplementation with folate in pregnancy. The results of laboratory quantification of 5-MTHF are presented in Table 1. The typical level of 5-MTHF was 300–400 µg per pill, with the exception of sample NUT20/F/2, which was an oral spray (liquid formulation) with a labeled dosage of 100 µg per spray and a labeled recommended use of four sprays daily in pregnancy (400 µg of folate). Only three samples (50%) were compliant with the European Commission (EC) guidance document in terms of acceptable tolerance in their content of food supplements (80–150%) [30]. One sample was considerably above the 150% margin, and two were below the 80% margin. Alarmingly, one of these did not contain a measurable level of 5-MTHF.

The content of 5-MTHF in one sample was above 1000 µg per dosage, exceeding the tolerable upper level (UL) for folate intake [43]. One of the key problems following high folate intake is the ability of folate to reverse megaloblastic anemia (related to vitamin B12 deficiency, which can have serious irreversible neurological consequences), thus delaying appropriate treatment with vitamin B12 [44]. There are also some concerns of possible adverse effects related to the development of cancer. While an adequate folate status is protective for cancer development, high intakes of folic acid might enhance cancer progression [45]; however, data on this are very limited.

On the other hand, one sample did not contain a quantifiable amount of 5-MTHF, despite the fact that the product was also intended for pregnant women (i.e., used a front-of-package health claim “essential vitamin for pregnancy”). This sample was subject to further analyses, but we were also not able to detect other authorized chemical forms of folate. As disclosed by market recalls in different EU countries, the manufacturer has suspended the production and sale of the product due to diminishing levels of folate [46,47]. It should be noted that 5-MTHF is known for its poor stability in comparison to other forms of folates [48]; however, the quality control of the manufacturer was not sufficient to identify this issue before releasing the product on the market. Considering that women of child-bearing age supplement their diets with folic acid to decrease the risk of neural tube defects [31,32], it is of particular importance that products marketed as a source of folate are actually a source of this vitamin. If this is not the case, this could also be regarded as a public health concern and not just a quality issue. Domínguez et al. [49] also very recently investigated quality of food supplements containing folate. They focused on products targeting pregnant women on the Spanish marketplace. They conducted the laboratory quantification of folate in four (*N* = 4) food supplements, and the analytical results were consistent with the declared values in all cases. However, it should be noted that only one of these products contained folate in form of 5-MTHF, while the other three supplements contained more stable pteroylmonoglutamic acid, which was not in focus of our study.

### 3.2. Food Supplements for Supplementation with Vitamin D in the General Population

**Figure 1:**
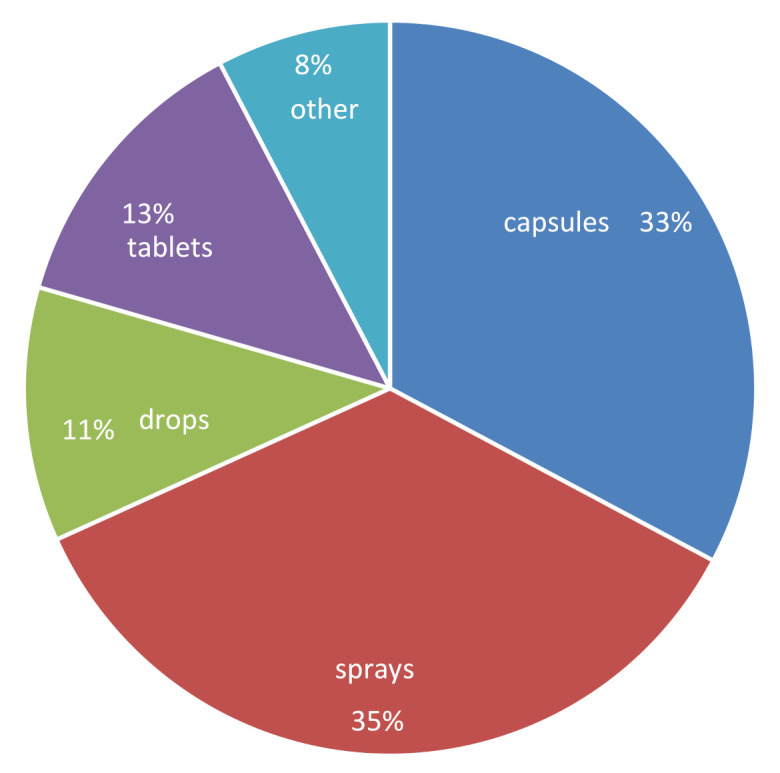
Frequency of usage of different formulations of vitamin-D-containing food supplements (Slovenia, 2020; *N* = 598).

To identify which products are most commonly used in Slovenia for supplementation with vitamin D, a survey involving 688 participants was conducted. Altogether, 90 participants (13%) reported supplementing vitamin D with subscription drugs (medicines). These were not in the scope of our study due to regulatory differences between food supplements (in the scope of EU food regulations) and medicines. Quality control standards used in the manufacturing of food supplements are, therefore, not comparable with medical GMPs. However, the majority of subjects (87%; *N* = 598) reported supplementation with vitamin D via food supplements. Various types of pharmaceutical formulations were reported, among which the most common were oral sprays (35%) and capsules (33%), followed by tablets (13%) and drops (11%) (Figure 1). Altogether, the use of 95 different food supplements was reported in the survey.

Twenty-four (*N* = 24) samples of vitamin-D-containing food supplements with the highest survey-reported usage were identified according to the sample selection protocol (described in Section 2.1.2), and they were purchased in local pharmacies, specialized stores, and online stores. Each of the selected samples was reportedly used by 3–46 (average: 6.4) respondents in our survey.

**Figure 2:**
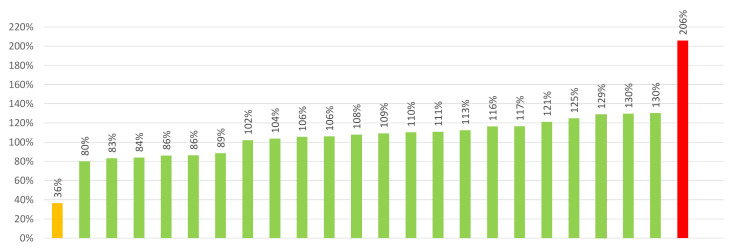
Analytically determined content of vitamin D in samples of food supplements as a percentage (%) of the content provided by manufacturer on labeling (*N* = 24). Each row represents one analyzed food supplement. Note: yellow (vitamin D content below the 80% of the labeled value); green: (80-150%); red: (above 150%).

The labeled vitamin D content in the sampled food supplements was in the range of 10–100 µg/dosage. In line with the sampling protocol, the most frequent content of vitamin D in supplements in our sample was 25 µg/dosage (*N* = 9). The majority of the samples (*N* = 17; 70%) were in the range of 15–62.5 µg/dosage, four (17%) were labeled with 10 µg, and three (13%) were labeled with 100 µg per dosage. The results of laboratory quantification of vitamin D in these samples are presented in Table 2 and Figure 2. With the exception of two samples, all others (*N* = 22; 92%) were compliant with the EC guidance document on acceptable tolerance in their content in food supplements (80–150%) [30]. One sample was above the 150% margin (NUT20/D/5; 206%), while one sample was considerably below the 80% margin (sample NUT20/D/4, 36%). This sample contained 9 µg of cholecalciferol instead of the declared 25 µg. These observations were in line with observations of other studies, which investigated content-related issues in vitamin D supplements in Europe [18,19,24]. We should also note that both of our samples that were outside the 80–150% of declared content level had an above-average number of users according to our survey (~270% above average).

As previously mentioned, our set of samples also contained three supplements with a labeled dosage of 100 µg of vitamin D, which is on the margin of the UL for the daily intake of vitamin D [43]. Hypercalcemia is one of the most concerning effects of vitamin D toxicity. It should be noted that in patients with vitamin D hypervitaminosis, hypercalciuria and a depressed parathyroid hormone (PTH) status can be normocalcemic [50], thus indicating that hypercalciuria and kidney stone formation might be earlier phases of hypervitaminosis [43]. In our study, the actual content of cholecalciferol in two of the samples with a labeled dosage of 100 µg of vitamin D was below the UL level but still in the range above 80% of the declared content. In one sample, laboratory analyses showed a content of vitamin D above 100 µg; however, with consideration of the extended uncertainty of the analytical method (with a coverage factor *k* = 2, corresponding to a probability interval of 95%), we could not exclude that the actual content was also within the margin of 100 µg. 

Considering that all samples were purchased, we also had their prices available. Across the sample, prices ranged from 0.01 to 0.42 EUR per 25 µg (1000 IU) of vitamin D, with a mean price of 0.15 EUR. The three products with the lowest price (both per 25 µg vitamin D and per recommended dosage by manufacturer) were formulated as oil drops. This can be explained by the fact that the manufacturing of oil drops is cheaper than the production of other formulations. Laboratory analyses (quantification of vitamin D) showed a sufficient quality of these cheapest food supplements. Interestingly, the most expensive sample (with price 0.42 EUR per daily dosage and per 25 µg vitamin D) was the only food supplement in our set where we found an insufficient vitamin D level (NUT20/D/4; 36% of the declared vitamin D content). This indicates that a higher price of a food supplement is not a guarantee of the quality.

### 3.3. Study Limitations

While we sampled all the relevant products in the case of supplements containing 5-MTHF, the number of vitamin D supplements was too high for a comprehensive analysis. Though we took a quite large sample of these food supplements, only samples with highest reported usage frequencies were included (*N* = 24). The strength of such an approach is that we covered most relevant products, but the limitation is that the quality of more niche food supplements might be different. Some strengths of the study are that we sampled food supplements independently of the manufacturers and that we purchased them on the market in the same way as typical consumers. However, in this way, we were unable to control some study parameters, such as the time from manufacturing to our analyses. Nevertheless, all analyzed food supplements were well within the declared expiry date. For example, the expiry dates of all samples with an insufficient quantity of vitamin were more than one year after the date of laboratory analyses. Additionally, we only investigated one production series of each product. Ideally, quality assessment should be done on different production series, but we should note that similar approach was also used in other studies [12,13,14,15,16,17]. Furthermore, only two types of vitamin-containing food supplements were investigated; therefore, the results cannot be generalized to other types of products.

## 4. Conclusions

The efficiency of nutraceuticals is affected by different complex parameters, including the bioavailability of the ingredient and the presence of other components, whether in the product itself or in the diet of the consumer. However, a basic predisposition for efficiency is that the nutraceutical contains a sufficient quantity of the active ingredient. This is related not only to the amount of the constituent added during manufacturing but also to its chemical stability. Due to the limited stability of many constituents of nutraceuticals, manufacturing is commonly done with excess in order to assure a sufficient quantity during the product’s shelf life. These challenges can lead to a considerable variability of the content of the active ingredient in products. On one hand, products could contain too low quantity of the active ingredient due to instability, so products are unable to affect the health status. On the other hand, nutraceuticals could contain more of their active ingredient than needed, which can present a health risk. We therefore investigated active ingredient content quality issues with two types of food supplements on the EU marketplace: products containing folate in the form of 5-MTHF and those containing vitamin D. Both of these active ingredients are essential for specific population groups; therefore, the low quality of such products presents a public health risk. This study showed a high variability in the content quality of the examined products. Though this was a case study that only investigated one production batch of each food supplement, we can conclude that while the majority of the investigated vitamin-D-containing supplements were within the tolerable level of content variability, this was not the case in products containing the less stable 5-MTHF. In both types of food supplements, we found samples with a very low vitamin contents (0% and 36% for 5-MTHF and vitamin D, respectively), as well as products with more than double quantity of the expected amount of the active ingredient. These results show the need for the better quality control of the food supplements. Food manufacturers need to assure sufficient quality control both in the manufacturing process and in products’ shelf lifetimes. Additionally, regular quality control measures for content should be conducted by the authorities. In the absence of regulatory conditions for good manufacturing practices, voluntarily quality control schemes would enable consumers to identify products of sufficient quality.

## Figures and Tables

**Table 1:** Results of laboratory quantification of 5-methyltetrahydrofolate (5-MTHF) in sampled food supplements and conformity with regulatory requirements.

Sample ID	Labeled Amount of 5-MTHF per Dosage ^a^	Analytically Determined Amount of 5-MTHF per Dosage	% Labeled Content ^b^	Comment ^c^
NUT20/F/1	400 µg	587 ± 6 µg	147%	
NUT20/F/2	100 µg	0 µg	0%	Below limit of quantification. Below 80% limit.
NUT20/F/3	400 µg	302 ± 13 µg	76%	Below 80% limit
NUT20/F/4	400 µg	587 ± 25 µg	147%	
NUT20/F/5	300 µg	290 ± 11 µg	97%	
NUT20/F/6	400 µg	1120 ± 35 µg	280%	Above 150% limit. Above UL level. ^d^

Note: ^a^ Folate in the form of (a) calcium-l-methylfolate or (b) glucosamine salt of (6*S*)-5-methyltetrahydrofolic acid. ^b^ Percentage (%) of the laboratory-determined content of 5-MTHF in comparison with the content provided by manufacturer on labeling. ^c^ According to European Commission guidance document [30], an acceptable content is within the interval of 80–150% of the labeled content. ^d^ The tolerable upper level (UL) for intake of folate is 1000 µg [43].

**Table 2:** Summary of results of laboratory quantification of vitamin D in sampled food supplements and conformity with regulatory requirements (*N* = 24).

Vitamin D ^a^ Content ^b^	*N*	%	Actual Range of Cholecalciferol	Comment ^c^
Below 80% of declared content	1	4%	36%	
80–150% of declared content	22	92%	80–130%	One sample was above the UL limit ^d^
Above 80% of declared content	1	4%	206%	

Note: ^a^ In all samples, vitamin D was added in the form of cholecalciferol. ^b^ Percentage (%) of the laboratory-determined content of cholecalciferol in comparison with the content provided by manufacturer on labeling. ^c^ According to European Commission guidance document [30], an acceptable content is within the interval of 80–150% of the labeled content. ^d^ The tolerable upper level (UL) for intake of vitamin D is 100 µg [43].

## Data Availability

The data presented in this study are available on request from the corresponding author.
